# Primary pyogenic ventriculitis caused by *Neisseria meningitidis*: case report and review of the literature

**DOI:** 10.1099/jmmcr.0.005078

**Published:** 2017-01-31

**Authors:** Firza Gronthoud, Ibrahim Hassan, Pippa Newton

**Affiliations:** ^1^​ University Hospital of South Manchester, Southmoor Rd, Wythenshawe, Manchester M23 9LT1, UK; ^2^​ University Hospital of South Manchester, Southmoor Rd, Wythenshawe, Manchester M23 9LT2, UK; ^3^​ National Aspergillosis Centre, 2nd Floor, Education and Research Building Hospital, Manchester, Wythenshawe M23 9LT, UK

**Keywords:** primary pyogenic ventriculitis, headache, neurologic symptoms, pleocytosis, drainage, antimicrobial treatment

## Abstract

**Background.** Pyogenic ventriculitis is a well-known complication of meningitis, brain abscesses and intraventricular drains. Primary pyogenic ventriculitis is a rare entity and few cases have been described so far. We report the first case of primary pyogenic ventriculitis in an adult caused by *Neisseria meningitidis* and present an overview of all reported adult primary pyogenic ventriculitis cases in the English literature.

**Methods.** A PubMed search was performed using the terms ependymitis, ventricular empyema, pyocephalus and ventriculitis. Filter was set for adults and English. Articles in which pyogenic ventriculitis was a complication of well-known risk factors were excluded. A total of five cases of primary pyogenic ventriculitis were identified.

**Results.** There were seven adult patients. Only one patient showed signs of meningeal irritation. Four patients had positive blood cultures with *Escherichia coli* (one patient), methicillin-resistant *Staphylococcus aureus* (one patient), one patient was bacteraemic with *Enterococcus faecalis*, *Escherichia coli* and *Peptostreptococcus* spp., and *N. meningitidis* (our patient). In four patients cerebrospinal fluid was sent for culture, which yielded methicillin-sensitive *Staphylococcus aureus* (one patient), *Peptostreptococcus* spp. (one patient), *Streptococcus intermedius* (one patient, identified via 16S PCR) and *Listeria monocytogenes* (one patient). Cerebrospinal fluid cell count was determined in four patients and showed pleocytosis in all four cases. Ventricular drainage was performed in four patients. Five patients survived.

**Discussion.** We report the first case of pyogenic ventriculitis caused by *N. meningitidis*. Primary pyogenic ventriculitis is a rare entity with various clinical presentations caused by various bacterial species. Treatment consists of adequate antimicrobial therapy, and ventricular drainage may be necessary.

## Abbreviations

CT, computed tomography; INR, international normalized ratio; MRI, magnetic resonance imaging.

## Background

Pyogenic ventriculitis is a recognized complication of meningitis, brain abscess, intraventricular surgery and presence of intraventricular drains. It has also been reported as a rare complication of endocarditis and urosepsis [1, 2]. There are no well-defined definitions for ventricular infections, and pyogenic ventriculitis has been variably referred to as ependymitis, ventricular empyema, pyocephalus or ventriculitis [3]. Primary pyogenic ventriculitis is rare with few cases described. We report the first case of a primary pyogenic ventriculitis in an adult caused by *Neisseria meningitidis*. We also present an overview of all adult primary pyogenic ventriculitis reported in the English literature.

## Case

A 55-year-old male, previously fit and well, presented acutely in our emergency department with fever, vomiting, swelling and erythema of his right foot and ankle and occipital headache without meningism or photophobia.

He had not recently travelled abroad and no weight loss or night sweats were recorded. He drank approximately 32 units of alcohol per week and was an ex-smoker with 40 pack years. Physical examination was unremarkable. Laboratory investigation showed C-reactive protein 332 mg l^−1^, white cell count 40.0 (neutrophils 38.9) × 10^9^ l^−1^, d-dimer 22 447 ng ml^−1^, international normalized ratio (INR) 1.4, lactate 2.5 mmol l^−1^ and platelets 139×10^9^ l^−1^.

A blood culture was taken and intravenous piperacillin/tazobactam 2.5 g t.i.d. was started. A computed tomography (CT) scan of his head could not differentiate between intraventricular blood and pus. Unfortunately, a lumbar puncture was not performed due to agitation and a raised INR. The following day magnetic resonance imaging (MRI) was performed which showed a ventriculitis and piperacillin/tazobactam was discontinued and intravenous ceftriaxone 2 g b.i.d. was started. A PCR for respiratory viruses was negative. His human immunodeficiency virus test was negative and immunoglobulin levels were normal. MRI of his right foot showed a tenosynovitis. The next day a venous blood culture was positive with Gram-negative diplococci in both aerobic and anaerobic bottles which subsequently were identified as *N. meningitidis* (API NH system) with the following MICs: penicillin 0.094 mg l^−1^, cefotaxime 0.003 mg l^−1^, rifampicin 0.023 mg l^−1^ and ciprofloxacin 0.008 mg l^−1^. Rifampicin was added for 1 week. The patient continued to show clinical improvement and was discharged home on outpatient antibiotic therapy to complete 6 weeks of treatment. He received a meningococcal vaccine (against groups A, C, W135 and Y). At a follow-up appointment, he had fully recovered with no neurological sequelae.

## Methods

We searched PubMed, EMBASE and the Cochrane Library using the search terms ependymitis, ventricular empyema, pyocephalus and ventriculitis. Filter was set for adults and Language. Exclusion criteria were: (1) ventricular drain-associated ventriculitis, (2) ventriculitis as a complication of meningitis, (3) ventriculitis as a complication of brain abscess, (4) complications of neurosurgical procedures and (5) trauma. Remaining articles were identified by screening titles and abstract and excluded if the diagnosis of ventriculitis was not clear, if the article was not available or if the study was written in a language other than English. We extracted data on patient characteristics, microbiology results, treatment and outcome.

## Review

### Study characteristics

A total of 488 articles were evaluated for eligibility. After screening of title and abstract, full texts of 55 articles were retrieved for evaluation. Most of them described ventriculitis secondary to the presence of an intraventricular drain or underlying condition such as meningitis of brain abscess. Five cases of primary pyogenic ventriculitis in adults were identified, all derived from case reports (Table 1).

**Table 1.** Summary of reported cases of primary pyogenic ventriculitis

Age	Gender	Symptoms	Meningism	Location	Underlying medical condition	Causative Agent	Sample	CSF pleocytosis	Antibiotic Treatment (days)	Neurosurgical treatment	Outcome	Ref
81	F	Right hemiplegia, aphasia	No	Left lateral ventricle	Unknown	*S. aureus*	CSF	Not performed	OXacillin (3), chloramphenicol (3)	No	Died	13
							Bloodculture not performed					
39	M	Fatigue, fever, rigors, frontal headache, nuchal rigidity, facial paresis, right extensor plantar response	Yes	Left lateral ventricle	Multiple dental extractions	*E. faecalis, E. coli, Peptostreptococcus* spp.	Bloodculture	Yes	Not documented	Ventricle drainage	Survived	4
						*Peptostreptococcus* spp.	CSF					
63	M	Headaches, feeling intermittently hot and sweaty, clumsiness, unsteadiness, diarrhoea and fever.	No	Right lateral ventricle	–	*S. intermedius*	CSF, 16s PCR	Yes	Cefotaxime (42), metronidazol (42)	EVD, later VP drain	Survived	14
							Bloodcultures negative		Rifampicin (14)			
62	M	Fever + headache after trip to Japan	No	Right lateral ventricle	Gastric cancer, total gastrectomy	*L. monocytogenes*	CSF	Yes	Vancomycin (8), ceftriaxone (8), ampicillin (?), gentamicin (?)	EVD	Survived	15
							Bloodculture negative					
66	M	Fever, anxiousness, restlessness, psychomotor retardation, generalized weakness and Suction-like mouth dyskinesias	No	Lateral ventricles	Laparoscopic left hemicolectomy, bowel obstruction and sclerosing peritonitis, renal failure	Methicillin Resistant *S. aureus*	Bloodculture, CSF	Yes	Vancomycin (5) followed by linezolid (49)	No	Survived	16
55	M	Fever, Intermittent occipital headache and right ankle swelling	No	Lateral ventricles	Hypertension, Hypercholesterolaemia, alcohol abuse and ex smoker (40 pack years)	*N. meningitidis*	Bloodculture	LP not performed due to raised INR	Piperacillin/tazobactam (1) and Clarithromycin (2) followed by ceftriaxone (41) and rifampicin (9)	No	Survived	Our case

### Patient characteristics

Patients were predominantly male with a median age of 63 years. Headache was present in four cases; only one patient showed signs of meningism. Apart from headache, four patients displayed neurological symptoms. Fever was reported in four patients.

### Microbiology results

Three patients had positive blood cultures with methicillin-resistant *Staphylococcus aureus* (one), *N. meningitidi*s (one) and one patient was bacteraemic with *Enterococcus faecalis*, *Escherichia coli* and *Peptostreptococcus* spp.

In five patients cerebrospinal fluid was taken yielding methicillin-sensitive *Staphylococcus* *aureus* (one), *Peptostreptococcus* spp. (one), *Streptococcus intermedius* (one, identified via 16S PCR), *Listeria monocytogenes* (1) and methicillin-resistant *Staphylococcus* *aureus* (1). Cerebrospinal fluid cell count was determined and all showed pleocytosis (*n*=4).

### Neuroimaging

In all patients diagnosis was made with neuroimaging. Interestingly, in four patients in whom both a CT scan and MRI was performed, the diagnosis was made by MRI whilst the CT scan was either normal or could not differentiate between blood and pus.

### Antibiotic treatment

Various antibiotic regimes were used. The route of administration was not reported in most cases. It is unknown if intraventricular administration of antibiotics was attempted in any of the cases found in the literature. Treatment duration was documented in four patients; three who survived had treatment duration between 42 and 49 days.

### Outcomes

Ventricular drainage was performed in three patients. Five patients survived, of whom three had ventricular drains.

## Discussion

Clinical manifestations of primary pyogenic ventriculitis are non-specific and the heterogeneity in organisms suggests various mechanisms by which a primary pyogenic ventriculitis could occur. One patient had recent dental extractions and blood culture showed a polymicrobial flora. In most of the patients a blood culture was positive, which suggests infection by haematogenous spread. A bacteraemia is probably not the only mechanism through which a ventriculitis occurs as reports of primary pyogenic ventriculitis are scarce. Based on clinical presentation and neuroimaging findings we excluded meningitis in our patient. Ventriculitis is a potentially fatal infection and early and accurate diagnosis with appropriate treatment is essential. Because clinical symptoms and signs are non-specific, the diagnosis relies mainly on neuroimaging.

A CT scan is less sensitive than MRI and the diagnosis can be missed as CT can show no abnormalities or can mislead the clinician to a diagnosis of intracerebral bleeding [4]. MRI is more reliable; irregular ventricular debris is the most characteristic finding and can be used to distinguish between pus and blood [5]. Hydrocephalus and ependymal enhancement are less frequent signs. In the early phases of infection diffusion-weighted imaging might be a useful MR sequence as conventional MRI may only show subtle changes [6].

A meningitis and ventriculitis can show the same clinical and biochemical features. In the absence of a suspicion of pyogenic ventriculitis, pleocytosis and a positive cerebrospinal fluid culture could then be misdiagnosed as meningitis, which in turn could lead to an increased risk of mortality. Persistent infection and therapeutic failure should prompt the clinician to suspect a ventriculitis, which might require ventricular drainage and possibly longer treatment duration.

Serotyping of the *N. meningitidis* strain showed it belonged to group B. After the introduction of the Men C vaccine in 1999, overall levels of invasive meningococcal disease have decreased and capsular group B strains accounted for the majority of cases. In September 2015, the 4CMenB vaccine was added to the routine UK immunization schedule and future reports will show its impact on the dynamics of meningococcal disease [7].

Even with neuroimaging the diagnosis can be missed or mistaken for a different disease process. We therefore find it difficult to conclude whether a primary pyogenic ventriculitis is a rare or underdiagnosed infection. Due to the small sample size and the nature of case reports it is difficult to draw definite conclusions regarding treatment. The most common cause of ventriculitis is an extraventricular drain infection. Antimicrobial treatment is targeted against *Staphylococcus* spp., Enterobacteriaceae and *Pseudomonas aeruginosa*. The choice of drugs depends on knowledge of local resistance patterns, ability to cross the blood–brain barrier and blood–cerebrospinal fluid barrier, adequate drug concentrations at the site of infection and route of administration [8]. Empirical therapy typically consists of intravenous vancomycin in combination with intravenous ceftazidime or meropenem. No randomized controlled trials exist regarding duration of antimicrobial treatment and decision to remove or retain the drain and no official recommendations exist. Management strategies thus far have been similar to management of central line-associated bloodstream infections [9]. Beer *et al*. have proposed an algorithm for the management of extraventricular drain-related ventriculitis [10]. Further clinical trials are needed to determine its effectiveness. Even if the meninges are inflamed, the penetration of intravenous vancomycin into cerebrospinal fluid is poor [11]. Few studies have been published assessing the pharmacokinetics and clinical utility of intraventricular administration of agents such as vancomycin and aminoglycosides [12].

This is the first report reviewing all cases of primary pyogenic ventriculitis published in the English literature. Because there are no well-defined definitions for ventricular infection it is difficult to ascertain whether previous reports of ventriculitis and meningitis described the same clinical entity.

We hope this report leads to an increased awareness and timely recognition of this potentially fatal disease. Future prospective studies are needed to establish the true incidence of primary pyogenic ventriculitis and its management.
